# Neuroscience-informed psychoeducation for addiction: a conceptual and feasibility study

**DOI:** 10.3389/fpsyt.2025.1527828

**Published:** 2025-02-12

**Authors:** Tara Rezapour, Kayla L. McLean, Elena Psederska, Khashayar Niki Maleki, Hamed Ekhtiari, Jasmin Vassileva

**Affiliations:** ^1^ Institute for Drug and Alcohol Studies, Virginia Commonwealth University, Richmond, VA, United States; ^2^ Department of Cognitive Psychology, Institute for Cognitive Science Studies (ICSS), Tehran, Iran; ^3^ Department of Cognitive Science and Psychology, New Bulgarian University, Sofia, Bulgaria; ^4^ Metacognium LLC, Austin, TX, United States; ^5^ Department of Psychiatry, University of Minnesota, Minneapolis, MN, United States; ^6^ Department of Psychiatry, Virginia Commonwealth University, Richmond, VA, United States

**Keywords:** neuroscience-informed psychoeducation, addiction, adolescents, neurocognitive, research domain criteria (RDoC), metacogntive

## Abstract

Over the past few decades, our understanding of substance use disorders (SUD) has been reshaped by evidence from neuroscience, which suggests that SUD are characterized by specific neuromarkers that transcend traditional diagnostic boundaries and act as pre-diagnostic markers that could be targeted through preventive attempts. Connectivity-based neuromarkers or brain networks have emerged as a promising framework, providing new insights into the neurocognitive mechanisms of SUD. Utilizing this data-driven framework assists prevention and intervention developers in offering a non-judgmental insight for adolescents regarding the potential vulnerability of neurocognitive systems to continued substance use. Given the importance of such awareness, this paper proposes a neural network-informed approach based on research domain criteria (RDoC) to characterize the content of neuroscience-informed psychoeducation designed for SUD. Furthermore, we argue that various features related to content and structure need to be considered when developing such interventions delivered through digital platforms (e.g., apps and websites). Finally, we introduce a theory-driven app called “NIPA”, developed with the aim of increasing adolescents’ awareness and resilience to the effects of drugs and other emotional triggers on brain and cognitive functions.

## 1 Introduction

Psychoeducation has been widely applied for different health related conditions (mental and medical disorders) in both clinical and community settings. Broadly speaking, psychoeducation is a process of teaching clients specific and general information about their illness and treatment (1). Specific information provides more detailed illness-related information, e.g., diagnosis and therapeutic trajectories, while general information includes more translational and general content, such as problem-solving skills and healthy life-style (1). Since the 1980s, when Anderson et al. introduced the term psychoeducation (2), translational research has reshaped the psychoeducational regimens and transferred findings from basic science to more practical level. By growing the knowledge of neuroscience in the context of mental illness, our understanding of risk factors, precursors, neural correlates, and therapeutic approaches has been much improved and the field of neuroscience-informed psychoeducation has emerged. “Neuroeducation”, “internal education”, “neuroscience literacy”, “neuroliteracy” and “Brain talk” are other relevant terms used to describe a class of interventions intended to educate individuals and reframe their perceptions of mental illness using neurobiological knowledge (3–5).

The application of psychoeducation in the treatment of substance use disorders (SUD) can be traced back to the 1940s, when alcoholism was acknowledged as an independent public-health problem and “*the mantle of stigma covered the subject”* (6). Early interventions targeted primarily treatment-seeking individuals who were at later stages of the addiction cycle characterizing severe SUD, rather than earlier stages such as substance experimentation or problem use (7, 8). Years later, psychoeducational interventions advanced by going beyond therapeutic interventions and getting applied as a tool for addiction prevention, particularly for children and adolescents. The first flashes of a psychoeducational program for children appeared in a commercial cartoon produced by the Partnership for a Drug-Free America (PDFA) in response to a cartoon that tricked kids to smoke. The PDFA’s cartoon featured the effects of drugs on the brain, depicting them as an egg dropping into a frying pan (9). Another example of such “Scare tactics” was the “Just Say No” campaign, known as Drug Abuse Resistance Education (D.A.R.E.), which aimed to teach students about the dangers of substance use by sending uniformed cops into the schools (10). These zero tolerance campaigns prevalent between the 1970s and 1980s were unsuccessful in reducing substance use behaviors.

Given the failure of such fear-based deterrence attempts, a new wave of educational approaches has emerged, characterized by non-judgmental and informative content that encourages students to make informed decisions about substance use (10). These educational approaches largely focused on knowledge and skill development in prevention, early intervention, and harm reduction formats. The knowledge includes information regarding the harmful effects of substance use and corrects normative expectations, while the skills training builds personal and social competencies, as well as establishes refusal skills (11). Accordingly, several successful educational interventions have been developed and tested in school settings; such as Red Frogs (12), Just Say Know Prevention Program (10), Unplugged (13), Life Skills Training (11), Project Towards No Drug Abuse (14), Reconnecting Youth (RY) (15), School Health and Alcohol Harm Reduction Project (SHAHRP) (16), Project ALERT (17) and ALERT Plus (18), Reasoning and Rehabilitation V2 (R&R2) program (19).

Over the past few years, interventions for adolescent have evolved from their initial focus on classic drug-related knowledge (e.g., the biological impact, including short- and long-term consequences of substance use, substance use standards, and prevalence) and skills training to leveraging adolescents’ curiosity about the brain’s involvement in substance use (20). The “*Seductive Allure of Neuroscience” (SANE)* (20) for adolescents has revealed a unique opportunity to integrate neuroscience knowledge with substance use prevention. The *SANE* proposes that psychological phenomena are more appealing and health messages more persuasive for adolescents when they are accompanied with brain-related information. For example, a recent harm reduction program, the ‘*Respect Your Brain’*, developed by Debenham and colleagues (2022), aimed to leverage neuroscience literacy of young people using a series of short animations (21). They found that watching brain-related animations could effectively engage people with the topics and positively change their attitudes towards the brain and substance use. Another program (22), which applied neuroscience to improve drug-based knowledge and teach practical coping skills is the ‘*Illicit Project’*. This program covered different aspects of the neuroscience of SUD in three modules including Alcohol and the Developing Brain; MDMA, Cannabis Use, Harm Reduction; and Mental Health and Wellbeing. Evidence from the pilot study indicates lasting effects (6 months) of the program on reducing drug and alcohol use compared to the control group who received health education. Therefore, it seems that knowledge about the role of the brain in the etiology and maintenance of SUD could lead to meaningful change in thoughts and behaviors (23). Moreover, given the high potential for learning and flexibility during adolescence, young people can benefit from preventive interventions to reduce the probability of SUD later in life (24, 25).

On the other hand, the rapid development of digital health technologies (e.g., mobile applications, websites), has introduced tremendous changes in the field of designing behavioral interventions and provides numerous opportunities for personalizing interventions and increasing their feasibility and accessibility. Digital platforms offer therapists and educators the chance to improve the quality of therapeutic and training services by incorporating unique features such as multimedia content, personalized feedback, and interactive elements. These platforms also allow services to be delivered affordably, regardless of time and location, which greatly enhances their scalability. Using digital health interventions also warrants the replicability and fidelity of the intervention and removes the barriers of differences in therapists’ skills and knowledge. Due to the popularity of digital health technologies, substantial progress has been made towards developing e-health preventive interventions (delivered via internet, computers, tablets, mobile technology, or tele-health) in the recent years (26–31). These interventions that typically offer a set of normative education and life skills training provide good empirical evidence and promising results about the efficacy of digital health in preventing or mitigating substance use in adolescents. Moreover, digital preventive interventions provide an opportunity for young users who prefer to remain anonymous during the training course and so reduce the stigma and embarrassment that they may perceive due to their needs for seeking help. This issue is complicated even further in adolescents, as they are highly impacted by stigma, and stigma can in turn create a major barrier to service seeking (32).

Acknowledging previous works, the present study aims to describe a conceptual framework for substance use prevention grounded in neuroscience knowledge and brain networks. Then, we propose key recommendations for developing a digital neuroscience-informed psychoeducation aimed at substance use prevention and introduce a sample educational app, termed ‘NIPA,’ as one of the novel tools developed to improve adolescents’ metacognitive awareness and enhance their resilience against SUD. Additionally, we provide preliminary feasibility and acceptability data regarding the app in a sample of college students and delineate the next steps for future studies.

## 2 Adolescents’ resilience against addiction: neural networks perspective

A large body of research has implicated the mechanisms involved in substance use vulnerability in adolescents. Family history, genetic and environmental factors, and neurodevelopmental adaptations are risk factors known to be associated with increased vulnerability in young people (33). Moreover, SUD is more probable in the presence of cognitive immaturities, particularly in memory and learning, goal-directed behaviors, decision making and impulse control which are common during adolescence (34, 35). According to the Dual Systems Model and the Maturational Imbalance Model, adolescent risk-taking results from a temporary imbalance between two neurobiological systems: the subcortical socioemotional system, which is responsive to emotion, reward, and novelty, and the prefrontal cognitive control system, which guides controlled action, planning, and decision-making (36). A key assumption of both models is that the socioemotional system (System 1) develops faster and earlier in adolescence than the cognitive control system (System 2). As a result, adolescents are presumed to be particularly vulnerable to high-risk behaviors and tend to pursue quicker rewards (e.g., instant pleasure, peer acceptance) (37, 38). Consequently, during adolescence neural networks may respond differently to emotionally salient stimuli, such as substances. For example, in a hypothetical scenario, a young person who is new to high school and unable to find close friends may be more vulnerable to experiment with drugs, in the hope of finding friends and being accepted by a group of peers. Initial recreational and exploratory substance use leads to neurobiological changes that contribute to repeated and problematic use. As a result of these alterations and subsequent neuroplasticity, brain networks show abnormal functional reorganizations, leading to disrupted cognitive functions. However, a balanced functioning of these two systems could underpin a trait termed *Resilience*.

**Figure 1:**
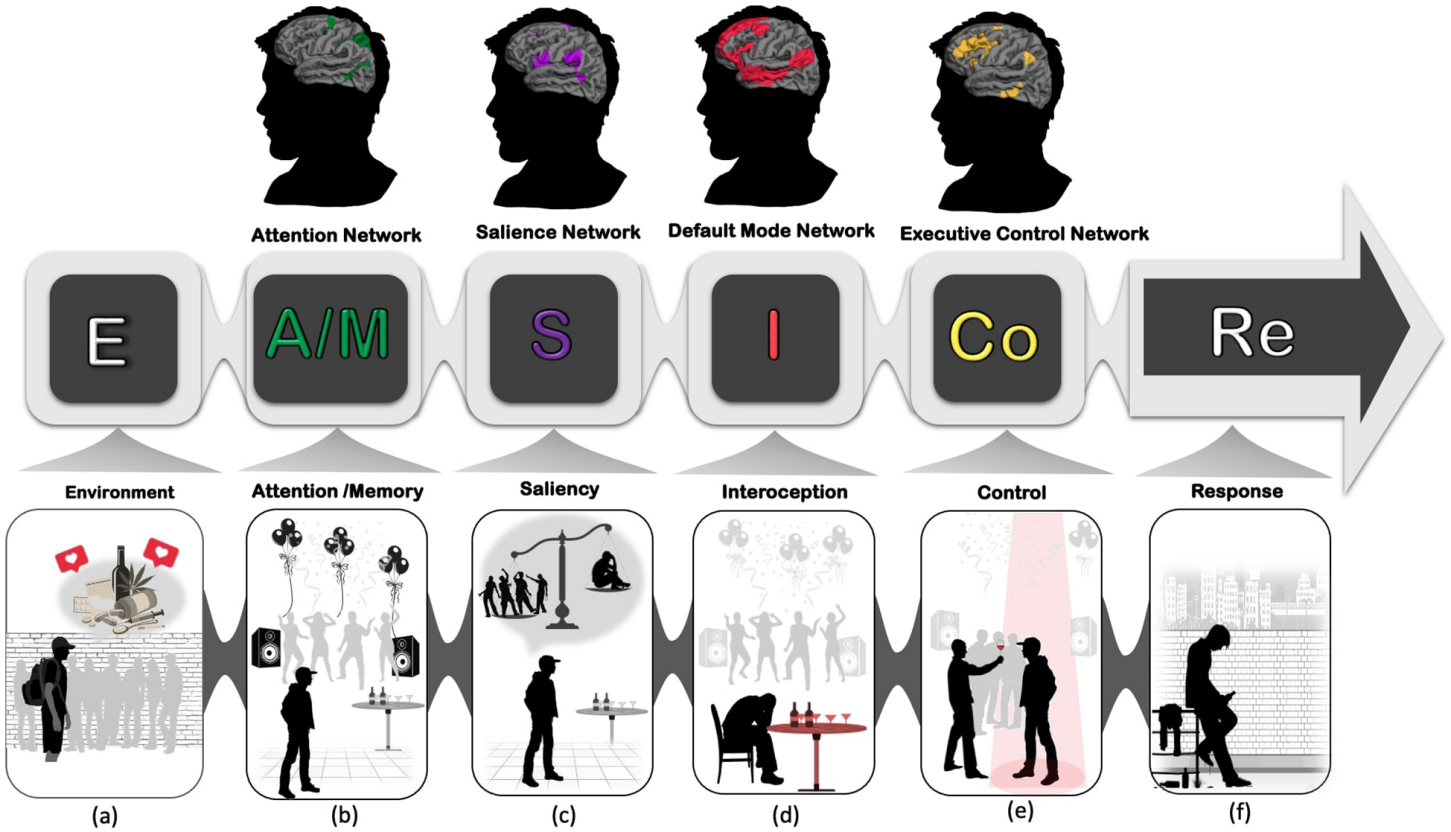
EASICoRe model as a neuroscience-informed conceptual framework, indicates different neurocognitive mechanisms involved in substance use vulnerability in adolescents. **(A)** Environment: Adolescents enter high school, experiencing increased peer pressure and exposure to environmental cues such as alcohol and drugs, along with a desire for social approval and being accepted into a group; **(B)** Attention/Memory: Gradually, adolescents’ attentional systems become biased towards drug/alcohol-related cues, and hedonic drug-related memories are consolidated in their memory; **(C)** Salience: Repeated exposure to these cues, leads the brain to overweight using drugs and alcohol as a prerequisite for remaining in the peer group over the fear of rejection and loneliness; **(D)** Interoception: Interoceptive signals such as heart rate and skin temperature are misinterpreted as a sign of urge to use drugs/alcohol; **(E)** Control: Executive control over inhibiting impulsive signals toward drug/alcohol use becomes increasingly challenging; **(F)** Response: Drug-taking behavior is reinforced by the hedonic pleasure experienced each time individuals use drugs/alcohol.

In general, resilient people are characterized by their ability to recover quickly after experiencing environmental risk or adversity and by adaptively coping with negative events and emotions. Resilience can be inferred from resistance to maladaptive behaviors when individuals face tragic or life-threatening events (39). A growing body of neuroscientific research aims to elucidate the brain networks underlying resilience and connect them with related cognitive processes (24, 40). However, while relatively few studies have focused on the specific brain networks associated with resilience or vulnerability against SUD, growing evidence highlights the importance of specific neural networks in the progression of SUD (25, 35). These networks include the Attention Network, Default Mode Network, Salience Network, and the Executive Control Network. Each of these networks is involved in specific cognitive functions and their balanced interactions could enhance one’s ability to overcome emotional adversity, and resolve the barriers in favor of positive outcomes. One of the conceptual models that we have suggested to explain the underlying neurocognitive mechanisms underlying resilience is the “EASICoRe” model (41). This conceptual model defines the major cognitive target processes involved in the dynamic response to drug-related cues. According to this model, once vulnerable individuals are exposed to an Environmental trigger (both internal and external cues), their Attentional resources are selectively allocated to process different aspects of the trigger (e.g., emotional, physical), while their Memory is biased toward recollecting relevant drug-related memories. By integrating the information from attention and memory, the evaluation system starts processing the Salience of incoming information and comparing them with subjective goals/values based on available appraisal schemas. At this moment, various somatic signals originate from within the body transferring information related to bodily experiences (e.g., heart rate, respiration rates). These Interoceptive signals contribute to emotional/appetitive experience and affect decision-making particularly under risk and uncertainty. Followed by evaluation, Inhibitory Control may be activated to control impulsive desires and habits and direct behavior Response toward more goal-oriented action. From the perspective of neuroscience, each of these cognitive functions is correlated with an activation of a distinct brain network. (**Figure 1**). In the following section, we describe these four networks and their dynamic interactive roles in building resilience against addiction.

### 2.1 Attention Network (AN)

Attention regulation refers to the process by which individuals control how their attention is allocated toward specific stimuli while ignoring irrelevant ones. This regulatory process is crucial for human survival, as our attentional resources are capacity-limited, requiring us to be selective when faced with competing information (42). Individuals can regulate their attention through two distinct but intertwined systems: the dorsal attention network (DAN) and the ventral attention network (VAN) (43). DAN, which involves the frontal eye fields (FEF), the superior parietal lobules (SPL), and the inferior parietal sulci (IPS), is responsible for goal-directed processing and top-down, voluntary attentional allocation. In contrast, VAN, thought to comprise the right temporoparietal junction (TPJ) and ventral frontal cortex (VFC), is involved in detecting unexpected, unattended, or salient stimuli. This latter network is responsible for bottom-up allocation and is known to be involuntary (44, 45). Poor attention regulation, exhibited by changes in the functional architecture of DAN and VAN, is prevalent among substance users (46), who often show reduced ability to deliberately switch their attention away from drug-related stimuli and to ignore the thoughts and emotions that tempt them to focus on drug use.

### 2.2 Default Mode Network (DMN)

Addiction is a pathological learning disorder in which many individuals with SUD are unable to successfully retrieve the experienced negative consequences of their past actions, reflect on them, and plan for a drug-free future (47). Findings from resting-state functional connectivity neuroimaging studies have identified the role of the Default Mode Network (DMN) in mental time travel, where individuals move back and forth between the past, present, and future to reflect on their autobiographical experiences and predict possible future situations. The DMN is also involved in self-awareness and interoceptive processes which allow individuals to accurately perceive signals received from internal organs (i.e., heart, skin, muscle, stomach) and interpret them as a sign of specific emotional state (i.e., anxiety, craving, fatigue) (48–50). This network involves three main components: a midline subsystem (including the medial prefrontal cortex, posterior cingulate cortex, and precuneus), a medial temporal subsystem (including the medial temporal lobe, medial parietal cortex, inferior parietal lobe, and ventromedial prefrontal cortex), and a dorsal medial prefrontal subsystem (including the dorsal medial prefrontal cortex, temporoparietal junction, and lateral temporal cortex) (51). Impaired internally-oriented cognition, including memory retrieval, mental imagery, and prospective thinking, may lead to increased vulnerability to continued substance use in adolescents. When vulnerable individuals are exposed to drug-related cues, they might fail to mentally imagine the consequences of their drug use based on previous memories and, as a result, may be unable to plan protective actions. Moreover, disruption of the DMN contributes to poor self-awareness and inaccurate interoception, which could lead to maladaptive responses, such as drug use, to regain homeostatic balance.

### 2.3 Salience Network (SN)

SUD is characterized by heightened salience attributed to any form of drug-related cues across various sensory modalities (e.g., the sight of a lighter, the smell of a cigarette, the sound of rolling) and by an expectation of greater reward from obtaining them (52). A set of brain regions including the amygdala, the anterior insula and the dorsolateral cingulate cortex are involved in detecting salient competing stimuli, processing reward, interoception, motivation, and emotion (53, 54). The other critical role of the SN is in risky decision making, in which individuals prefer smaller immediate rewards rather than larger but delayed ones (55). Disrupted activity of the SN is associated with increased impulsivity and emotional reactivity. Lack of premeditation before using drugs is an example of impulsivity related to the activity of the SN.

### 2.4 Executive Control Network (ECN)

Compromised executive functions such as response inhibition, attention, working memory, planning, problem solving and flexibility, are known to be key cognitive factors involved in substance use initiation and maintenance (34). Impairments in these functions are associated with the ECN, a task-oriented brain network underlying various cognitive processes involved in decision-making and self-regulation (48). The ECN includes the dorsolateral prefrontal cortices, dorsomedial prefrontal cortex, inferior parietal lobule, anterior–superior posteromedial cortices, and medial temporal gyrus (56). One of the key functions of the ECN that is often impaired in substance users is cognitive flexibility. In clinical and experimental studies, poor flexibility is manifested by perseveration, characterized by the failure to change strategies in accordance with feedback (57).

According to the EASICoRe model, vulnerable adolescents become hypersensitive to drug-related cues (SN), fail to control their attentional resources in favor of ignoring these cues (DAN, VAN), exhibit weakened inhibition to regulate their behavior against substance use (ECN) and, ultimately, are less sensitive and less able to recall the experienced negative consequences of using drugs (DMN). In addition to their individual effects, these networks can interact with each other, yielding more complex phenotypes. For example, impaired decision-making in substance users could be due to many different reasons associated with aberrant functioning in distinct neural networks. It could be related to attentional and memory difficulties in remembering previous choices or consequences of actions (DAN, VAN). It could also be related to motivational mechanisms, which bias choice behavior such as hypersensitivity or hyposensitivity to reward and punishment (SN), or to cognitive control and/or cognitive flexibility difficulties (ECN), evidenced by perseveration on substance use despite potentially fatal negative consequences. These complex phenotypes may portend different biotypes of SUD and other psychiatric disorders which, in turn, may be targeted by more personalized interventions (58–61).

Therefore, neuroscience can inform adolescents about how continued substance use may alter brain functions and structures through a simplified and comprehensive educational format. To make such interventions more engaging and interactive, digital platforms could offer various opportunities to support this translation. In the following section, we discuss this novel approach in the field of prevention.

## 3 Digital neuroscience-informed psychoeducation for addiction prevention

Providing educational tools about substance use and addiction to adolescents is challenging, as they tend to be more influenced by their peers’ beliefs and may find it difficult to trust educational content, which traditionally tends to be stigmatizing and fear-based. The growth of the field of addiction neuroscience is a novel and promising path that helps prevention scientists translate the complex science of the brain into comprehensible materials. This brain-derived education that capitalizes on the SANE (20) phenomenon particularly when delivered via a digital platform, captivates younger audiences and facilitates dialogue between them and scientists who view addiction as a brain disorder rather than a moral or criminal issue (62). To facilitate the development of such digital educational intervention, we summarize the most important considerations in the following section in terms of content and structure.

### 3.1 Content

#### 3.1.1 Science-delivered education

Adolescents trust more educational content which is evidence-based, non-stigmatizing and simple to comprehend. Using brain-based education that focuses on brain structure and function seems to be non-judgmental and engaging for young people. Within therapeutic settings, growing evidence suggests that providing patients with biological explanations of their disorders can reduce self-stigmatizing attitudes and potentially remove the mental barriers to recovery (23).

#### 3.1.2 Familiarity

To persuade adolescents about the potential harm of drug use on the brain, symptoms should be explained in terms of concrete and tangible examples (e.g. vignettes), rather than abstract concepts, and clarified with illustrations of how they might interfere with daily functioning. For instance, how attention instability could affect productivity in studying.

#### 3.1.3 Gamified cognitive exercises

Besides the educational facts and conceptual information regarding cognitive functions, offering cognitive games to increase individuals’ insight into the mechanisms behind these functions is particularly useful. Once individuals play these games, they can clearly understand how specific cognitive functions may be engaged both in performing the game and in activities of daily living.

#### 3.1.4 Applied cognitive strategies

The feasibility of the provided brain strategies is evaluated through their applicability in the context of real life. Convenient, available, and simple strategies may be perceived as meaningful and relevant, encouraging individuals to adopt and maintain them in their daily lives.

### 3.2 Structure

#### 3.2.1 Length of training session

A key factor in developing educational content, particularly for digital platforms, is the length of the training sessions. Due to our limited attention span, educational sessions that exceed 20-25 minutes might not contribute to efficient learning (63). Therefore, it is suggested to break down the length of the educational sessions into shorter episodes, separated by breaks or entertaining activities.

#### 3.2.2 Multimedia content

Integrating various content modules, including animations, cartoons, text, games, videos and music, could enhance the learning experience and user engagement. For example, using a game-based approach to design brain exercises may be more suitable for adolescents and young adults, who are generally active game players and more familiar with such contents (64). Using cartoons could also strengthen the effectiveness of key messages as cartoons grab attention, facilitate memory, amplify self-awareness, boost self-affirmation, and provide useful decision-making heuristics and means for emotion regulation and mental time travel (8).

#### 3.2.3 Self-assessment

A key phase in the learning process is self-evaluation. Educational interventions aimed at enhancing individual knowledge may have more lasting effects by allowing users to assess their own knowledge and learning (65).

#### 3.2.4 Language

Using concise, digestible language and simple metaphors of well-known concepts such as “*spotlight*” for “*attention*”, could help bridge the gap between science and practice for adolescents. This language can facilitate engagement with the content while introducing scientific terminology, and allow young people to discuss addiction without any resistance. They can also use this shared language to talk with their counselor or therapist to have more effective communication (23).

## 4 NIPA example: neuroscience-informed psychoeducation for addiction

Based on the theoretical background on the role of neural networks in substance use vulnerability among adolescents, a mobile application referred to as the ‘Neuroscience-Informed Psychoeducation for Addiction (NIPA)^1^ was developed as a metacognitive awareness program to promote resilience in the face of emotional triggers, particularly drugs and alcohol. The program delivers psychoeducational content in four 20-minute-long sessions, including neuroscience-based education and cognitive games and training. Once individuals install the application on their mobile devices, the first session is unlocked, granting access to the full content. All sessions follow a similar structure, which includes the following sections:

### 4.1 Introduction (Knowledge)

Each session begins with an animation depicting a specific cognitive problem (attention and concentration, memory, flexibility and inhibition, impulsivity and decision making). Subsequently, individuals are asked about their personal experience with the cognitive problem(s).

### 4.2 Games (Practice)

Following the introduction and exploration of specific cognitive functions and difficulties, individuals play the first two levels of a game (levels 1-2), which engages the specific cognitive processes reviewed in that session. The games are designed to raise individuals’ awareness of how they use specific cognitive functions to solve game-based scenarios. For instance, after watching an animation illustrating how attention difficulties can interfere with daily tasks, individuals play a hidden objects game, applying their attention to find target images in crowded backgrounds. After the neuroscience-informed educational section (described below), individuals repeat the game with an increased level of difficulty (levels 3-4), this time with greater awareness of the specific cognitive processes involved.

### 4.3 Neuroscience-informed psychoeducation (Knowledge)

This animated section aligns with the previous ones and explains specific cognitive functions implicated in SUD and their underlying brain networks (DAN/VAN, DMN, SN, ECN). For example, different types of attention, including sustained, selective, divided, and flexible attention, as well as voluntary and involuntary attention, are described in the first session, and the dorsal and ventral attention networks are introduced as their underlying neural networks. Moreover, immediately after playing the second round of games, individuals are provided with further scientific evidence on how different brain regions within a network are activated to invoke a cognitive function (**Table 1**). In this section, individuals also learn about specific threats to brain functions as a result of using drugs, alcohol, and other emotional triggers. For example, in the first session, attentional bias is introduced as a result of the disruption of the normal functioning of voluntary and involuntary attention. This section is presented through engaging cartoons and animations.

**Table 1:** Samples of narrations describing specific cognitive functions implicated in addiction and their underlying brain networks in the NIPA program.

Session	Cognitive Function	Introduction of the Brain Network	A Highlight related the Effects of Substances on Neurocognitive Functions
1	Attention	“There are certain regions in the brain named as the intraparietal sulcus, the Middle Temporal Area, and the frontal eye fields, which are formed the Voluntary Attention Network (VAN) and activated when we are hyper focused on a detail that helps you meet a specific goal. For example, those regions light up if you are on the highway looking for a specific exit sign, or when you are shopping and focused on finding that long red dress. In both cases, you are focusing on something specific to meet a goal …”	“Drugs and alcohol don’t just deliver hangovers, they can screw up your ability to control attention. They can cause Attentional bias–a fancy term which means your brain is drawn to drug-related cues in your environment like a super magnet. Not only do things that look like paraphernalia grab your attention like a pit bull on steroids, it doesn’t let go easily. It gets stuck in the reruns and it’s hard to redirect your attention to anything else…”
2	Memory	“When you recall your past personal memories or think about things that may happen in the future, a neural network called the Default Mode Network (DMN), gets activated in your brain. This network is vital for your sense of self. It drives self-reflection and is activated when you daydream. It’s the DMN that enables your mind to travel through time and think about the future and the past, sometimes even at the same time. DMN consists of different regions including the Medial prefrontal cortex, inferior parietal lobe, and lateral temporal cortex.…”	“When people first start using, it makes them feel so euphoric it carves a pathway of strong associations between drug use and feelings of pleasure. As a result, your brain gives drug-related cues a promotion in the motivational hierarchy, which impairs learning for non-drug related cues and makes drug-related memories more vivid, real, and worst of all–tempting. Drug use also enhances drug-dependent learning.…”
3	Cognitive flexibility and Inhibitory Control	“An example of inhibitory control occurs in real-life situations is when we have to inhibit automatic reactions we have developed (e.g., snap at loved ones) to change our behavior. The neural network that plays a critical role in this situation is named the Executive Control network (ECN). The ECN network includes frontoparietal brain regions such as the dorsolateral prefrontal cortex (dlPFC), the posterior parietal cortex, and the dorsal anterior cingulate cortex. …”	“Evidence from a number of studies shows that drug users show inflexibility in changing their behavior even in the face of negative and harmful consequences. This process is called Perseveration, which is characterized by uncontrollable inability to interrupt a particular behavior or to shift from one strategy or procedure to another, regardless of one’s goals and despite potential negative consequences…”
4	Decision Making and Impulsivity	“The neural network that plays a critical role in salience (value) attribution is called “Salience Network (SN)”. This network includes regions such as the Inferior frontal gyrus and temporoparietal junction that are displayed in purple. This network is important for assigning value to external or internal stimuli and directing our attention to detect and process them. When one is being impulsive and trapped in a narrow temporal window (e.g., thinking only a day ahead), this network attributes higher value to the immediate rewards at the expense of long-term goals…”	“Certain things, like drugs and alcohol, can interfere with how we assign value (salience attribution) to different things in our environment, and impair decision making and impulse control. Drugs and alcohol make it more difficult to ignore emotional stimuli and control our impulse to react immediately. When under the influence, people tend to make important decisions without considering their long-term consequences or their pros and cons…”

### 4.4 Brain training Strategies (Skills)

The final section of each session is dedicated to providing four specific cognitive training strategies to boost the specific cognitive functions reviewed in the session, aiming to improve individuals’ resilience when exposed to drugs and other emotional triggers. Each strategy is accompanied by an exercise where individuals are required to apply the strategy they have learned. For example, in the first session, mindfulness, deep breathing, and focused reading and listening skills are learned and practiced through exercises.

### 4.5 Wrap-up (Practice)

Once individuals complete each session, they are provided with session highlights as a conclusion and are then directed to a multiple-choice exam. This section is designed to improve the learning experience and consists of 4-5 questions. Immediately after completing the quiz, they receive feedback and scores.

Throughout all the sessions, we used different comic characters, including ‘Mr. Brain,’ to narrate complex brain-based concepts and add a sense of humor to make the content more engaging.

## 5 Pilot study to test the feasibility and acceptability of NIPA

To investigate the feasibility and acceptability of NIPA, we conducted a pilot study with a sample of 85 undergraduate students (Mean age =19.09 years; Female=85%). Participants for the current study were included from an ongoing longitudinal cohort study of college students at a large, urban, mid-Atlantic public university. This study was approved by the university’s review board (HM20018784) and all participants provided informed consent. For a detailed review of study methods see (66). Participants were invited by email and screened for eligibility. Inclusion criteria included (1) being an undergraduate student age 18 or older (2); previous experience/use of alcohol, and/or tobacco, and/or cannabis, and/or other drugs; and (3) being willing and able to download the app and complete the program. Eligible participants were asked to complete a set of self-report assessments (e.g., Barkley Deficits in Executive Functioning Scale, Monetary Choice Questionnaires). Study data were collected and managed using REDCap electronic data capture tools (67, 68).

**Figure 2:**
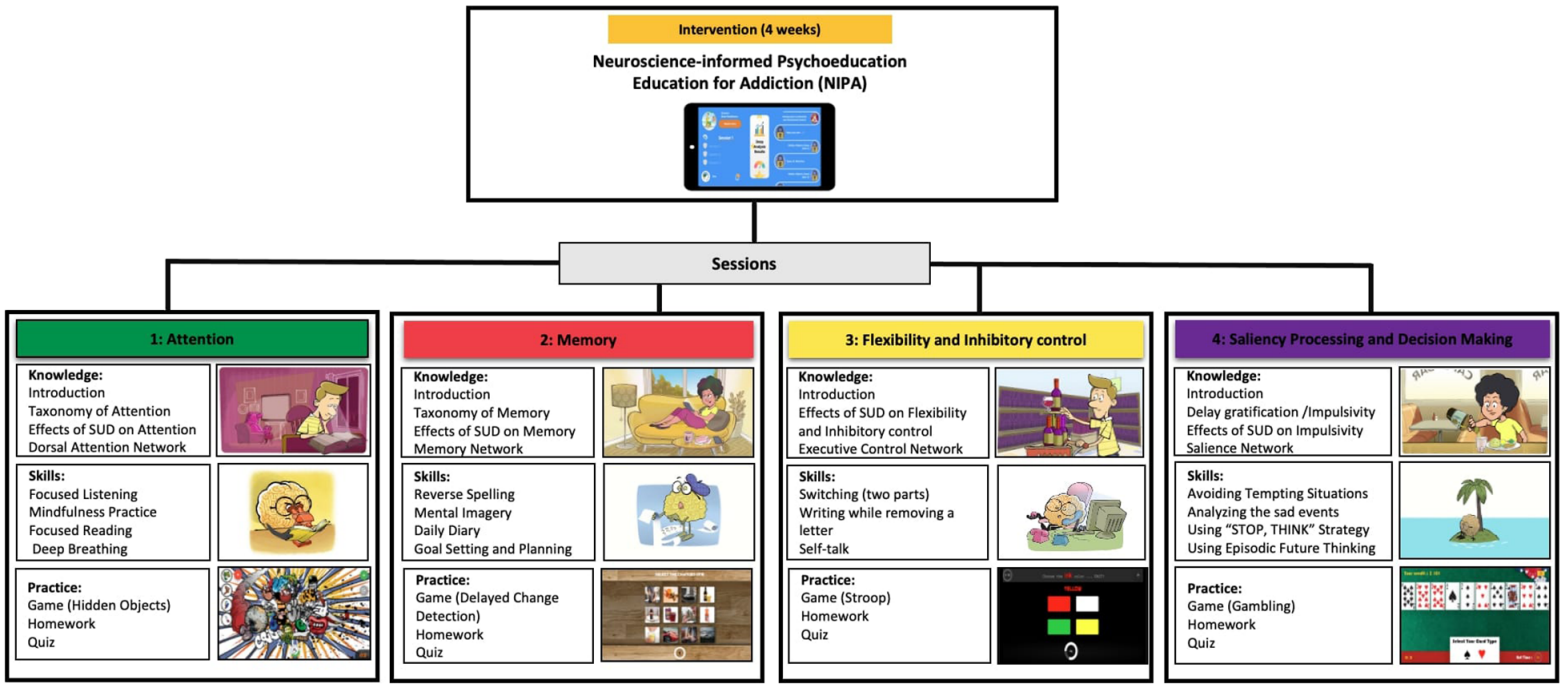
The architecture of the NIPA intervention, including cognitive domains (Attention, Memory, Flexibility, Decision Making), knowledge (Neuroscience-informed psychoeducation), skills (Brain training strategies), and practice (Games and quiz) designed in 4 sessions.

Once participants completed the pre-intervention baseline assessments, they started the NIPA program and after the final session of the intervention, they completed post-intervention assessment, which included feasibility and acceptability measures to evaluate the utility of NIPA as an educational program (**Figure 2**). The present paper reports the feasibility results, whereas the outcomes of the pre- and post-assessments are reported in another study.

### 5.1 Attendance and completion of training sessions

We invited 100 students enrolled in the cohort project to participate in the study. Eighty-five participants expressed interest to participate and completed the pre-intervention assessment. Of these, 72 completed one session, 71 - two sessions, 69 - three sessions, and 68 - all four sessions, all of whom gave post-intervention feedback.

### 5.2 Acceptability measures

The acceptability questionnaire included 10 items rated on a 7-point Likert scale. The questionnaire covered eight areas including (1) perceived enjoyment [*How much did you enjoy using the app*]*?* (2), convenience [*How easy did you find to install and use the app*]*?* (3), perceived informativeness [*How informative did you find the information provided about the brain and addiction*]*?* (4), applicability [*How much do you think the brain training strategies could be applicable to your daily routine*]*?* (5), perceived effectiveness [*How effective do you think the app might be*]*?* (6), continued use [*Would you like to continue using the app*]*?* (7), recommendation to peers [*Would you like to recommend the app to your friend*]*?*. The last three questions were related to the overall program [*How satisfied are you with the number of sessions? How satisfied are you with the session length? Overall, how satisfied are you with the program*]*?*. Participants were also asked to identify their most and least likable part of using the app as well as the biggest barriers they encountered for completing the sessions. **Table 2** shows the results from students’ perceived acceptability measures of the NIPA program.

**Table 2:** Feasibility and acceptability scores for the NIPA program.

Area of acceptability	Mean score (out of 7)
How much did you enjoy using the app?	5.04
How easy did you find to install and use the app?	6.39
How informative did you find the information provided about the brain and addiction?	5.81
How much do you think the brain training strategies could be applicable to your daily routine?	4.96
How effective do you think the program might be?	5.04
Would you like to continue using the app?	4.18
Would you like to recommend the app to your friends?	4.43
How satisfied are you with the number of sessions?	5.34
How satisfied are you with the session length?	5.31
How satisfied are you with the program?	5.60

According to participants’ rating, the games were the most (70.8%) and the brain training strategies were the least (33.8%) likable sections of using the app. The participants also identified the brain training strategies section as the biggest barrier to continuing using the app.

## 6 Discussion

As the age of drug use initiation decreases, interventions targeting the delay or reduction of problematic substance use among young people have expanded significantly. Preliminary forms of such interventions focused on reducing supply, reducing or delaying drug demand, and limiting the health and social harmful effects of substance use by criminalizing it (69). Although these classical approaches showed promise in reducing substance use by providing general knowledge about its negative consequences and teaching certain resistance skills, they appear to be ineffective in raising individuals’ awareness of the critical role of the brain in preventing or maintaining substance misuse. This kind of neuroscientific approach to reshaping the understanding of addiction as a brain disorder rather than a criminal justice issue, is an emerging field that seems to be more persuasive and acceptable among young people.

This new approach simplifies complex concepts about how different substances affect the brain, how neurocognitive mechanisms underlie the development of addiction, and how the brain can build resilience against SUD. Additionally, it could help adolescents better understand their own cognitive health. This is particularly important for adolescents with undiagnosed psychiatric disorders, such as ADHD. Parents and teachers of these youth might report behaviors such as leaving tasks incomplete, struggling to enjoy activities, and making impulsive decisions. Unaware of these symptoms and associated cognitive dysfunctions, adolescents become vulnerable to more serious challenges in daily life, such as a lack of persistence in pursuing long-term goals and being easily distracted by impulsive thoughts or risky behaviors such as substance use). Therefore, it seems necessary to provide adolescents with applied neuroscientific knowledge that can increase their understanding and awareness of cognitive functions, the long-term consequences of substance use on the brain, and evidence-based strategies to build resilience and improve brain health.

Moreover, framing drug-related education with brain-based language provides a safe learning environment for adolescents, where they don’t feel labeled or stigmatized for their attitudes toward substance use. The efficacy of such an intervention could increase even further when delivered through a digital platform, which is a salient communication tool for young people. Digital platforms have a number of benefits that can increase the efficacy of prevention and intervention efforts, such as: (1) higher anonymity compared to in-person sessions; (2) highly reproducible manner of intervention delivery; (3) by being available 24/7, they can provide ‘on demand’ therapeutic services when individuals need them most; (4) reduce stigma by diminishing the potential for public exposure; and (5) high scalability – increased access at low cost. Given the importance of expanding digital neuroscience-informed psychoeducation interventions for substance use prevention, we developed NIPA as a novel mobile app designed to inform adolescents about the neurocognitive mechanisms of addiction. It is noteworthy that NIPA uses a network perspective to discuss these neurocognitive processes affected by substance use, as brain networks provide a more accurate understanding of cognitive functions compared to other levels of analysis. This is supported by recent neuroimaging studies that focus on brain network analysis to represent the complex functional interactions between different regions underlying specific cognitive processes (70, 71).

Results from this pilot study support the feasibility and acceptability of NIPA among college students. We found that the brain games were the section liked the most by our participants, while the brain training strategies were rated lower compared to other sections. The mean scores for different measures of acceptability, including enjoyment, perceived convenience, informativeness, applicability, effectiveness, continuity, recommendation to peers, and satisfaction, were relatively good. Moreover, we received some qualitative feedback on various aspects of the program, which will be used to revise and modify subsequent versions of the app. These changes may help ensure that the content is more persuasive and acceptable for adolescents and emerging adults, enabling it to influence their awareness of the harmful effects of drugs on the brain and their intention to use or continue using them.

We wish to acknowledge a few limitations of our study. First, the small sample size and the lack of a control group reduce the generalizability and comparability of the results. Second, our sample was comprised predominantly of females with only a few male participants, which restricted our ability to examine gender differences in the analysis. Third, to improve learning and facilitate effective consolidation of the content, we are currently developing homework assignments to be completed between the sessions. Despite these limitations, our study introduces a novel psychoeducational tool in the field of addiction prevention for adolescents. Our findings support the feasibility of such neuroscience-informed interventions among adolescents in a college setting. Moreover, this app program has the potential to be used as a preventive tool for high school students and as a harm reduction intervention for adolescents who recreationally use drugs and alcohol. Additionally, that, due to its simple and comprehensible content, the app could be integrated into the therapeutic course of SUD treatment to raise patients’ awareness about the importance of seeking cognitive training interventions. However, further research is needed to determine whether using the app can change individuals’ attitudes and intentions toward drug use, as well as improve cognitive outcomes such as decision-making and impulsivity in each of these contexts.

## Data Availability

Data from this study are available via dbGaP (phs001754.v4.p2) or via spit4science@vcu.edu to qualified researchers who provide the appropriate signed data use agreement.

## Footnotes

1 This application is available under the name of Metacognium in App Store and Google Play.
